# Evaluating the correlation between pretreatment ^18^F-FDG PET/CT metabolic parameters and tumor-infiltrating lymphocyte levels in nonluminal breast cancer and impact on survival

**DOI:** 10.3389/pore.2024.1612014

**Published:** 2025-01-08

**Authors:** Muge Tamam, Halim Ozcevik, Gamze Kulduk, Merve Nur Acar Tayyar, Gunduzalp Bugrahan Babacan

**Affiliations:** ^1^ Department of Nuclear Medicine, Prof. Dr. Cemil Taşcıoğlu City Hospital, University of Health Sciences, Istanbul, Türkiye; ^2^ Department of Nuclear Medicine, Hamidiye Medical Faculty, University of Health Sciences, Istanbul, Türkiye; ^3^ Department of Pathology, Prof. Dr. Cemil Taşcıoğlu City Hospital, University of Health Sciences, Istanbul, Türkiye

**Keywords:** tumor-infiltrating lymphocytes, nonluminal breast cancer, tumor heterogeneity indices, FDG, metabolic parameter

## Abstract

**Background and Objectives:**

This study aims to evaluate the correlation between Tumor-Infiltrating Lymphocyte (TIL) levels and Fluorine-18 fluorodeoxyglucose (^18^F-FDG) metabolic parameters, including spleen and bone marrow FDG uptake and tumor heterogeneity in non-luminal breast cancers (NLBC), and to elucidate their association with survival outcomes.

**Methods:**

We retrospectively analyzed data from 100 females with stage 2–4 NLBC who underwent pretreatment ^18^F-FDG Positron emission tomography-computed tomography (PET/CT). TIL was scored based on Hematoxylin-Eosin-stained specimens and ^18^F-FDG PET metabolic parameters, including maximum standardized uptake value (SUVmax), mean standardized uptake value (SUVmean), metabolic tumor volume (MTV), total lesion glycolysis (TLG), liver, spleen, and bone marrow FDG uptake were calculated. Heterogeneity Index (HI)1, HI2, and HI3 indices were analyzed with FDG metabolic parameters. The association between these factors and overall survival was analyzed using multivariate Cox regression models.

**Results:**

TIL showed weak negative correlations with tumor size, tumor (T), and metastasis (M) stages. No significant correlation was found between TIL levels and overall SUV values. However, in stage 4, TIL correlated positively with liver, spleen, and bone marrow SUV values and negatively with heterogeneity indices (HI2, HI3). Higher tumor size, HI values, and Bone marrow-to-liver ratio (BLR) SUVmean were associated with increased mortality. A TIL cut-off value of <5 was linked to significantly worse survival.

**Conclusion:**

Our study demonstrates a strong connection between TIL, FDG metabolic parameters, and tumor heterogeneity, particularly in advanced NLBC. Although TIL is not generally associated with SUV values, its association with certain metabolic and heterogeneity indices suggests that it is important in influencing survival. Further research involving larger cohorts and diverse breast cancer subtypes is needed to validate these results.

## Introduction

Non-luminal breast cancers (NLBC), including human epidermal growth factor receptor-2 (HER2) positive and triple-negative breast cancers (TNBC), are characterized by aggressive clinical behavior and poor clinical outcomes [1, 2]. Effective management necessitates a thorough understanding of tumor biology and the interaction of various prognostic variables [3].

Tumor-infiltrating lymphocytes (TILs), immune cells seen in the tumor microenvironment, have been identified as important prognostic indicators in non-luminal breast cancers [1, 4, 5]. Several clinical trials have reported the prognostic and predictive importance of TILs in breast cancer. High TIL levels often indicate a robust immune response, which has been linked to a better prognosis and improved treatment outcomes [3, 6]. Higher Tumor-Infiltrating Lymphocytes have been associated with a better prognosis in both HER2-positive and TNBC patients, indicating a more favorable immunological milieu that may increase responses to targeted therapies and correspond with better clinical outcomes and survival rates [7, 8]. Despite these associations, the variations in clinical outcomes based on differing TIL levels still need to be explored [9].

In addition to TIL, positron emission tomography-computed tomography (PET/CT) imaging of fluorine-18 fluorodeoxyglucose (^18^F-FDG) metabolic parameters provides useful information on the tumor’s metabolic activity [10–12]. FDG uptake in the spleen and bone marrow on PET/CT is also being recognized as a good biomarker of systemic immune response and tumor-related metabolic alterations [11, 13, 14]. These measures represent the tumor’s level and extent of glucose metabolism, quantifying its aggressiveness and metabolic burden [15].

Another critical factor, tumor heterogeneity, adds complexity to the management of breast cancer [16]. Heterogeneity can manifest in genetic, phenotypic, and microenvironmental variations, influencing tumor behavior and response to treatment [6, 16–18]. Integrating FDG metabolic parameters with tumor heterogeneity assessments can reveal metabolic variability patterns crucial for understanding the tumor’s complexity and tailoring treatment strategies [8]. Tumor-infiltrating lymphocytes play a significant role in the immune response against breast cancer, influencing the tumor microenvironment and heterogeneity.

Understanding the relationships between TIL levels, FDG metabolic parameters, and tumor heterogeneity is essential for refining prognostic assessments and guiding personalized treatment strategies in non-luminal breast cancer [10, 12]. Evaluating the correlation between TIL levels and FDG metabolic parameters could improve predictions of overall survival [19].

This study aims to evaluate the correlation between TIL levels and FDG metabolic parameters, including spleen and bone marrow uptake and tumor heterogeneity, in nonluminal breast cancers and examine their association with survival outcomes.

## Methods

### Study design and patients’ selection

The records of 100 females with histopathologically confirmed newly diagnosed stage 2–4 nonluminal breast cancer who received pretreatment ^18^F-FDG PET/CT between June 2016 and September 2023 were reviewed retrospectively. Patients were assessed for pretreatment staging before beginning oncological or surgical treatment. All patients were initially diagnosed with core needle tru-cut biopsy. The study excluded patients with bilateral breast cancer, non-invasive ductal cancer histopathology, secondary primary malignancy diagnosis, pregnancy, male patients, stage 1 disease, insufficient clinical data, immunohistochemical data, no TIL rate, and no follow-up. Following the application of the exclusion criteria, the remaining patients were included in the research.

The patients were staged using the 8th edition of the American Joint Committee on Cancer (AJCC) Tumor-Node-Metastasis (TNM) system, which provides a complete classification for breast cancer based on tumor size (T), lymph node involvement (N), and the existence of distant metastasis (M) [20]. Tumor staging was evaluated using MR and PET/CT scans. The nodal staging was determined by analyzing the imaging data, physical exam, and sentinel lymph node biopsy results. Since staging with ^18^F-FDG PET/CT has no additional value in clinical stage I (T1N0) patients, the group of nonluminal breast cancer patients with stages 2–4 was included in the study.

Patient data was obtained from patient files, the hospital information management system, and Picture Archive and Communication Systems (PACS). Overall survival (OS) was determined by calculating the months between the date of diagnosis and the date of death registered in the death notification system (DNS). All patients underwent at least 1 year of follow-up.

### Pathological analysis

Nonluminal breast cancer includes the HER2-positive group, which is marked by high HER2 expression and the absence of ER and PR, and triple-negative breast cancer, which lacks expression of ER, PR, and HER2 [2].

The immunohistochemistry (IHC) for HER2 was performed using HER2 neu (EP3) monoclonal antibody. HER2 status was determined based on recommendations by The American Society of Clinical Oncology/College of American Pathologists (ASCO/CAP) guidelines [21]. Tumors were classified according to this guideline as positive (score:+3), equivocal (score:+2), and negative (score:+1/0). The equivocal category was tested by silver *in situ* hybridization (SISH) to determine whether there was overexpression of HER2 protein. The Ki-67 proliferation rate was counted in hotspot areas, with the percentage of tumor cells showing nuclear Ki-67 positivity being reported.

TIL was scored using the standardized method Products by the International Immuno-Oncology Biomarker Working Group on Breast Cancer [3]. TIL is defined as mononuclear cells that infiltrate tumor tissue. The scoring of TILs was based on Hematoxylin-Eosin (H-E) stained specimens and described as a percentage of the area occupied by TIL per total stromal area. The TIL score of the breast cancers was classified as low (<10%), intermediate (10%–40%), and high (>40%) by a pathologist blinded to the remaining clinical information. Only the index tumor was analyzed in this study if patients had multiple tumors.

### ^18^F-FDG PET/CT image technique

All patients underwent PET/CT after fasting for at least 6 h and then had their blood glucose levels checked. A serum glucose level was measured below 200 ng/dL, and an intravenous injection of 8–12 mCi (296–444 MBq) (approximately 8.1 MBq of FDG per kilogram of body weight) ^18^F-FDG was administered. Whole-body PET/CT imaging was performed on a biograph (Siemens Biograph 6, Chicago, IL, United States) using a full-ring high-resolution (HI-REZ) LSO PET and a six-slice CT scanner (Siemens Biograph 6, Chicago, IL, United States). Firstly, a non-enhanced CT scan was performed with the following parameters: 40–60 mAs, 140 kV, and 5-mm section thickness. Positron emission tomography scanning with 3 min per bed position was then acquired on the identical transverse field of view in the caudocranial direction. PET image datasets were reconstructed iteratively using the ordered subset expectation maximization algorithm with CT-based attenuation correction.

### ^18^F-FDG PET/CT image analysis

A General Electric Advantage Workstation (AW workstation Volume Viewer 3 software, version 3.2 (AWS) GE Healthcare, Waukesha, WI, United States) was used for all image analysis. The program was used to segment lesions automatically. Images were localized by starting evaluation with maximum intensity projection (MIP) images. Pre-segmentation of the lesions was performed by two Nuclear Medicine physicians. The lesions were marked with the help of auto-contour ROI (auto-contour ROI), and measurements were performed in all three planes (coronal-axial-sagittal) after it was determined that the entire lesion was within the ROI and that there were no other foci showing FDG uptake outside the lesion.

The tumor’s maximum standardized uptake value (SUVmax), mean standardized uptake value (SUVmean), peak standardized uptake values (SUVpeak), Metabolic Tumor Volume (MTV), and Total Lesion Glycolysis (TLG) were noted. Using the formula [injected dose (MBq) body weight (g)], SUVmax was determined based on body weight. The tumor contours were semi-automatically identified to determine MTV by employing a threshold of 42% of the SUVmax within the lesion. The SUVmean was multiplied by MTV to compute TLG. A volumetric zone of interest was defined to encompass the primary tumor completely. On checking the sagittal and coronal images, the volumetric region of interest border was semi-automatically adjusted if the volume extended beyond the borders of the primary lesion.

The SUVmax and SUVmean values for the liver, spleen, and bone marrow were calculated. Bone marrow SUV measurements were taken from the L2-L4 vertebrae. Subsequently, the following ratios were calculated: the mean SUV of the spleen to the mean SUV of the liver (SLR SUVmean), the SUVmax of the spleen to the SUVmax of the liver (SLR SUVmax), the SUVmax of the bone marrow to the SUVmax of the liver (BLR SUV max), and the mean SUV of the bone marrow to the mean SUV of the liver (BLR SUV mean).

Heterogeneity Index (HI) values and other continuous variables were analyzed: the lesions’ highest standardized uptake values (SUVmax) and peak standardized uptake values (SUVpeak) were recorded. The mean SUV (SUVmean) and minimum SUV (SUVmin) values at the threshold value of 30% were determined over SUVmax. Based on these values, the HI1 value was calculated using the formula (SUVmax-SUVmin)/SUVmean [16].

To calculate other HI values, metabolic tumor volume (MTV) and total lesion glycolysis (TLG) values were determined over the 40% threshold value of SUVmax. Then, the slope of the linear regression line of the MTV values at the threshold values of 30%–40%-50% over the new MTV values obtained by changing the threshold percentage to 30% and 50% values were calculated as HI2 [17].

With the same technique, the slope of the linear regression line obtained with the percentage change of MTV at 40% and 50% threshold values by accepting the MTV at 30% threshold value as 100% was determined as HI3 [18].

### Statistical analysis

Statistical Package for Social Sciences version 25.0 (SPSS, IBM Corp. Armonk, NY, USA) for Windows was used for statistical analysis. Descriptive statistics were presented as counts and percentages for categorical variables and mean, standard deviation, minimum, maximum, and median for numerical variables. The proportions between groups were compared using the Chi-square test. Comparisons of numerical variables between groups were performed using the Student's t-test to see if the normal distribution condition was met or the Mann-Whitney U test to see if it was not. Relationships between numerical variables were examined using Spearman Correlation Analysis, as the parametric test conditions were unmet. Determinant factors were analyzed using Linear Regression Analysis and Cox Regression Analysis. Findings that were statistically significant in univariable analysis were included in the multivariable backward model. Receiver operating characteristic (ROC) Curve Analysis was performed for the TIL cut-off value. Survival analyses were conducted using Kaplan-Meier Analysis. The alpha significance level was accepted as p < 0.05.

Ethical approval: The research was authorized by the decision of the Ministry of Health Istanbul, Türkiye Prof. Dr. Cemil Taşcıoğlu City Hospital Local Ethics Committee, numbered 254, dated 07.11.2023 (2023/254). The study was conducted according to the ethical principles in the Declaration of Helsinki and the guidelines of Good Clinical Practice/Good Laboratory Practice standards.

## Results

**FIGURE 1:**
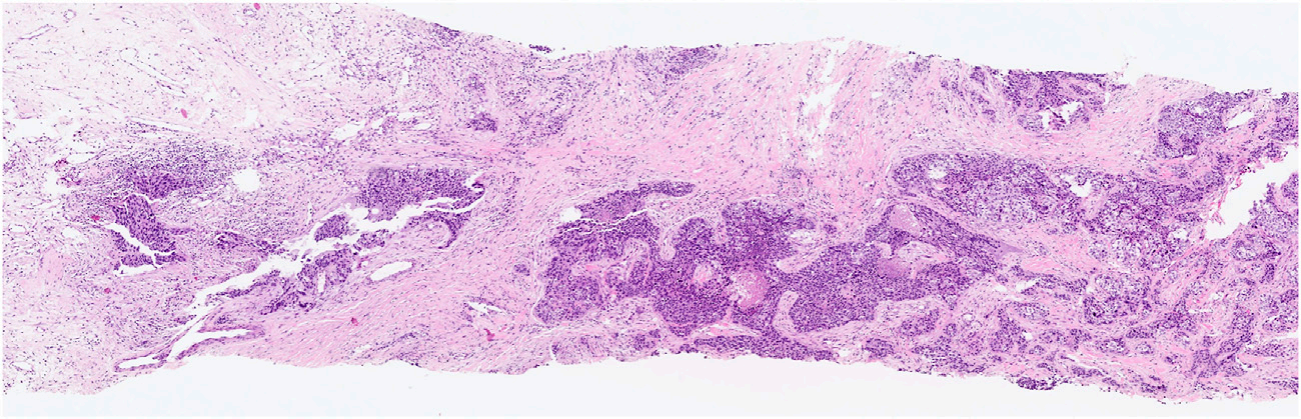
Low-grade TILs: a few lymphocytes in stromal tissue surrounding the cancer.

**FIGURE 2:**
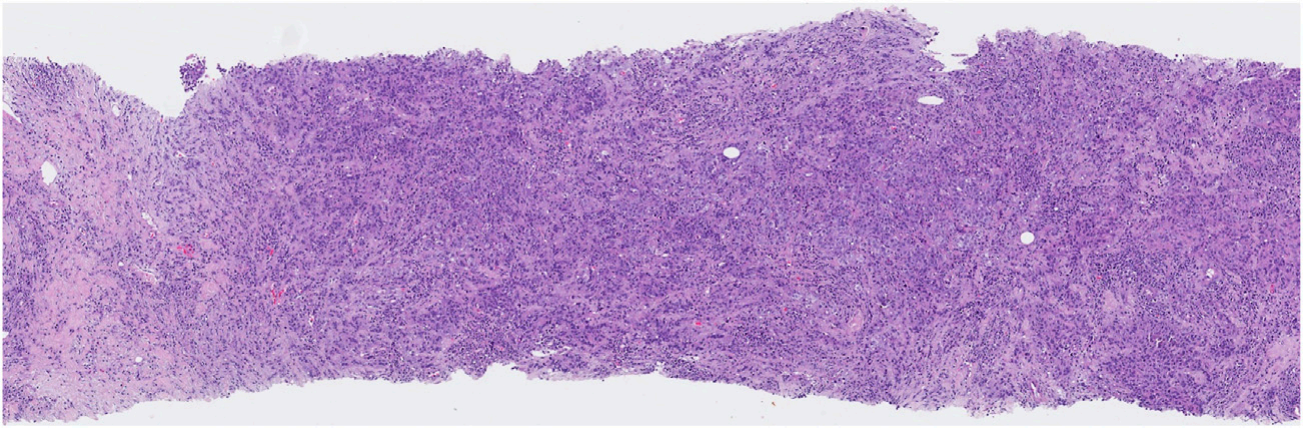
High-grade TILs: numerous lymphocytes are present in stromal tissue adjacent to the cancer.

The study included one hundred women with breast cancer. The patients’ mean/median age was 50.48 ± 11.54 (range 27–86). The HER2-positive BC group comprised 39 patients, while TNBC included 61. According to staging data, the disease was in stages 2 for 40 patients, 3 for 35, and 4 for 24 people. Two patients were in T1, 68 in T2, 20 in T3, and 10 in T4 tumoral stages, while 26 were in N0, 26 in N1, 19 in N2, and 29 in N3 nodal staging groups. Twenty-four patients had metastatic disease. The patient characteristics are summarized in Table 1. Of the 100 breast cancer lesions, 11 (11%) were classified as high TIL, 27 (27%) as intermediate TIL, and 62 (62%) as low TIL (Figures 1, 2). TIL had a negative and statistically significant association with tumor size and metastasis (p = 0.016 p = 0.047 p = 0.023) (Table 2). There was no statistical association between TIL levels and SUV values in non-luminal breast cancer.

**TABLE 1:** Patient characteristics.

		Total		Alive		Exitus		P^a^
		n	%	n	%	n	%	
TNBC/HER2	HER2	39	39.0	33	44.0	6	24.0	0.076
	TNBC	61	61.0	42	56.0	19	76.0	
Breast side	R	52	52.0	37	49.3	15	60.0	0.355
	L	48	48.0	38	50.7	10	40.0	
Pathologic Diagnosis	IC	5	5.0	3	4	2	8.0	0.455
	IDC	95	95.0	72	96.0	23	92.0	
Nuclear Grade	2	5	5.0	4	5.3	1	4.0	1.000
	3	95	95.0	71	94.7	24	96.0	
TILs	0–10	62	62.0	43	57.3	19	76.0	0.145
	10–40	27	27.0	24	32.0	3	12.0	
	>40	11	11.0	8	10.7	3	12.0	
T	1	2	2.0	2	2.7	0	0.0	**0.033**
	2	68	68.0	56	74.7	12	48.0	
	3	20	20.0	12	16.0	8	32.0	
	4	10	10.0	5	6.7	5	20.0	
N	0	26	26.0	23	30.7	3	12.0	0.151
	1	26	26.0	19	25.3	7	28.0	
	2	19	19.0	15	20.0	4	16.0	
	3	29	29.0	18	24.0	11	44.0	
M	0	76	76.0	66	88.0	10	44.0	**<0.001**
	1	24	24.0	9	12.0	15	56.0	
Stage	2A	25	25.0	24	32.0	1	4.0	**<0.001**
	2B	16	16.0	11	14.6	5	16.0	
	3A	14	14.0	14	20.0	0	0.0	
	3B	4	4.0	3	6.0	1	4.0	
	3C	17	17.0	14	18.7	3	12.0	
	4	24	24.0	9	3.7	15	62.3	
Multicentricity		30	30.0	22	29.3	8	32.0	0.801
Axillary Lymph Node positive		74	74.0	52	64.0	22	80.0	0.137
>Follow-up Median (Min-Max)		34 (9–97)		39 (10–97)		21 (9–76)		**<0.001** ^b^

^a^ Chi Square Test.

^b^ Mann Whitney U Test HER2, Human epidermal growth factor receptor-2; TNBC, triple-negative breast cancer; TILs, tumor-infiltrating lymphocytes; T, tumor; N, lymph node; M, metastasis.

**TABLE 2:** Correlation between TIL and all other parameters in all groups.

	TILs	
	r	p^a^
Ki67	0.141	0.161
Diameter	−0.241	0.016
T	−0.199	0.047
N	−0.100	0.322
M	−0.228	0.023
Stage	−0.147	0.145
SUVmax	0.037	0.717
SUVmin	−0.014	0.891
SUVmean	0.047	0.640
HI1 (max-min/mean)	−0.042	0.682
MTV %42	−0.154	0.125
TLG	−0.116	0.251
SUVpeak	0.039	0.697
HI2 (MTV threshold index)	−0.192	0.056
HI3 (% HI2)	−0.194	0.054
Liver SUVmax	0.061	0.545
Liver SUVmean	0.111	0.271
Spleen SUVmax	0.132	0.189
Spleen SUVmean	0.162	0.108
BM SUVmax	0.072	0.479
BM SUVmean	0.142	0.158
SLR SUVmax	0.017	0.867
SLR SUVmean	0.041	0.686
BLR SUVmax	−0.009	0.927
BLR SUVmean	0.082	0.418

^a^ Spearman Correlation Analysis.

TILs, tumor-infiltrating lymphocytes; T, Tumor; N, lymph node; M, Metastasis; SUVmax, maximum standardized uptake value; SUVmin, minimum standardized uptake value; SUVmean, mean standardized uptake value; HI, Heterogeneity Index; MTV, metabolic tumor volume; TLG, total lesion glycolysis; SUVpeak, peak standardized uptake value; SLR, spleen-to-liver ratio; BLR, bone marrow-to-liver ratio.

TIL was observed to have a positive correlation (p = 0.027 p = 0.046) with both Liver SUVmax and SUVmean in stage 3 patients. In stage 4, TIL levels were found to have a negative, statistically significant correlation with HI2 and HI3 and a positive, statistically significant correlation with Liver SUVmean, Spleen SUVmax, SUVmean, and Bone Marrow SUVmax, SUVmean (p = 0.023, p = 0.004, p = 0.049, p = 0.004, p = 0.004, p = 0.007, p = 0.009) (Table 3).

**TABLE 3:** Correlation between TIL and Stages with all other parameters.

	Stage 2		Stage 3		Stage 4	
	TILs		TILs		TILs	
	r	p	r	p	r	p
Ki67	0.194	0.229	0.187	0.275	0.173	0.419
Diameter	−0.348	0.028	0.015	0.932	−0.364	0.081
T	−0.333	0.035	0.012	0.946	−0.231	0.278
N	0.063	0.700	−0.165	0.336	−0.360	0.084
M			−0.182	0.288	−0.210	0.324
SUVmax	0.012	0.940	0.046	0.788	0.097	0.653
SUVmin	−0.056	0.731	0.072	0.677	−0.060	0.780
SUVmean	0.016	0.923	−0.010	0.956	0.178	0.405
HI1(max-min/mean)	−0.091	0.575	0.043	0.803	0.023	0.915
MTV %42	−0.055	0.735	0.050	0.772	−0.404	0.050
TLG	−0.071	0.664	0.037	0.830	−0.206	0.334
SUVpeak	−0.014	0.934	0.089	0.607	0.166	0.439
HI2	−0.152	0.350	0.047	0.786	−0.462	0.023
HI3 (% HI2)	−0.052	0.751	−0.090	0.602	−0.563	0.004
Liver SUVmax	−0.261	0.104	0.367	0.027	0.270	0.202
Liver SUVmean	−0.161	0.322	0.335	0.046	0.406	0.049
Spleen SUVmax	−0.177	0.276	0.277	0.102	0.567	0.004
Spleen SUVmean	−0.139	0.393	0.311	0.064	0.560	0.004
BM SUVmax	−0.222	0.169	0.090	0.602	0.539	0.007
BM SUVmean	−0.119	0.466	0.236	0.167	0.523	0.009
SLR SUVmax	−0.034	0.837	0.090	0.602	0.038	0.860
SLR SUVmean	−0.048	0.771	0.113	0.511	0.149	0.487
BLR SUVmax	−0.077	0.637	−0.116	0.501	0.242	0.254
BLR SUVmean	−0.023	0.889	0.099	0.564	0.328	0.118

^a^ Spearman Correlation Analysis.

TILs, tumor-infiltrating lymphocytes; T, tumor; N, lymph node; M, Metastasis; SUVmax, maximum standardized uptake value; SUVmin, minimum standardized uptake value; SUVmean, mean standardized uptake value; HI, Heterogeneity Index; MTV, metabolic tumor volume; TLG, total lesion glycolysis; SUVpeak, peak standardized uptake value; SLR, spleen-to-liver ratio; BLR, bone marrow-to-liver ratio.

TIL correlated positively with Ki67 at low levels, negatively with MTV%42, and adversely with metastasis in HER2-positive patients (p = 0.015, p = 0.045, p < 0.001). In TNBC patients, low TIL levels were observed to be inversely linked with tumor growth, HI1, and HI3 (Table 4).

**TABLE 4:** Correlation of TILs with all parameters in HER 2 and TNBC groups.

	HER2		TNBC	
	TILs		TILs	
	r	p	r	p
Ki67	0.388	0.015	−0.042	0.747
Diameter	−0.239	0.143	−0.256	0.046
T	−0.264	0.104	−0.217	0.093
N	−0.192	0.240	−0.043	0.744
M	−0.560	<0.001	−0.084	0.520
Stage	−0.277	0.088	−0.061	0.639
SUVmax	0.000	1.000	−0.004	0.977
SUVmin	−0.035	0.830	0.015	0.911
SUVmean	0.001	0.995	0.053	0.687
HI1(max-min/mean)	0.187	0.254	−0.377	0.003
MTV %42	−0.323	0.045	−0.122	0.350
TLG	−0.194	0.237	−0.107	0.410
SUVpeak	−0.055	0.738	0.015	0.908
HI2(MTV threshold index)	−0.216	0.187	−0.202	0.119
HI3 (% HI2)	−0.018	0.911	−0.333	0.009
Liver SUVmax	−0.153	0.351	0.156	0.231
Liver SUVmean	−0.054	0.746	0.152	0.242
Spleen SUVmax	0.036	0.830	0.173	0.183
Spleen SUVmean	0.101	0.542	0.202	0.119
BM SUVmax	−0.136	0.408	0.116	0.374
BM SUVmean	−0.024	0.883	0.143	0.271
SLR SUVmax	0.027	0.872	−0.008	0.952
SLR SUVmean	0.135	0.412	−0.012	0.929
BLR SUVmax	−0.117	0.478	−0.010	0.937
BLR SUVmean	−0.003	0.983	0.020	0.881

^a^ Spearman Correlation Analysis.

TILs, tumor-infiltrating lymphocytes; T, Tumor; N: lymph node; M, Metastasis; SUVmax, maximum standardized uptake value; SUVmin, minimum standardized uptake value; SUVmean, mean standardized uptake value; HI, Heterogeneity Index; MTV, metabolic tumor volume; TLG, total lesion glycolysis; SUVpeak, peak standardized uptake value; SLR, spleen-to-liver ratio; BLR, bone marrow-to-liver ratio.

The tumor size, HI1, HI2, HI3, and BLR SUVmean ratio were found to be statistically significantly higher in patients with shorter survival (p = 0.040 p = 0.048 p = 0.007 p = 0.013 p = 0.012) (Table 5).

**TABLE 5:** Univariate analysis to examine the risk effect of TILs on exitus.

		p	HR	%95 CI	
Enter Method	TNBC1HER20	0.382	2.898	0.267	31.424
	Stage (Ref:2)	0.000			
	Stage 3	0.239	3.172	0.465	21.647
	Stage 4	<0.001	69.19	11.33	422.3
	Axillary lymph nodes positive	0.833	0.848	0.182	3.945
	Diameter	0.198	1.036	0.982	1.093
	SUVmin	0.647	0.815	0.340	1.955
	SUVmean	0.588	0.863	0.507	1.470
	HI1	0.232	0.057	0.001	6.215
	MTV42	0.149	0.951	0.887	1.018
	TLG	0.766	1.001	0.995	1.007
	HI2 MTV threshold index	0.391	1.175	0.813	1.697
	HI3HI2	0.046	3.366	1.023	11.076
	Liver SUVmean	0.366	1.406	0.671	2.943
	BLR SUV max	0.839	1.450	0.041	51.816
	BLR SUV mean	0.456	4.646	0.082	264.150
	TILs	0.688	1.006	0.977	1.036
Backward Method	Stage (Ref:2)	0.000			
	Stage 3	0.223	2.434	0.581	10.190
	Stage 4	<0.001	32.88	9.086	118.99
	SUVmin	0.006	0.764	0.631	0.925
	HI3HI2	0.011	2.448	1.230	4.870
	BLR SUVmean	0.043	4.593	1.052	20.055

HER2, Human epidermal growth factor receptor-2; TNBC, triple-negative breast cancer; SUVmax, maximum standardized uptake value; SUVmin, minimum standardized uptake value; SUVmean, mean standardized uptake value; HI, Heterogeneity Index; MTV, metabolic tumor volume; TLG, total lesion glycolysis; BLR, bone marrow-to-liver ratio; TILs, tumor-infiltrating lymphocytes.

In the model created using variables identified with p < 0.250 in univariate analyses, the backward method revealed that Ki67, MTV %42, and multicentricity were statistically significant factors (p = 0.017, p = 0.030, p = 0.011) (Table 6).

**TABLE 6:** Multivariate linear regression analysis.

		p	Beta	B	%95 CI	
Model	Fixed			18.883	−4.871	42.637
	Ki67	0.186	0.145	0.129	−0.063	0.320
	Stage	0.793	−0.028	−0.290	−2.482	1.903
	MTV %42	0.310	−0.168	−0.130	−0.384	0.123
	HI2(MTV threshold index)	0.755	−0.051	−0.353	−2.598	1.892
	HI3 (% HI2)	0.145	−0.155	−4.012	−9.437	1.412
	Spleen SUVmax	0.162	−0.445	−14.051	−33.866	5.765
	Spleen SUVmean	0.116	0.488	18.578	−4.681	41.837
	BM SUVmean	0.427	0.114	2.729	−4.072	9.531
	TNBC1 HER2 0	0.441	0.094	3.887	−6.084	13.859
	Multicentricity	0.052	−0.202	−8.881	−17.831	0.069
	R Square	0.213				
Backward Method	Fixed			12.685	2.619	22.751
	Ki67	0.017	0.239	0.212	0.039	0.385
	MTV %42	0.030	−0.218	−0.169	−0.322	−0.017
	Multicentricity	0.011	−0.248	−10.902	−19.266	−2.537
	R Square	0.138				
Dependent Variable: TILs						

MTV: metabolic tumor volume; HI: heterogeneity index; SUVmax: maximum standardized uptake value; SUVmean: mean standardized uptake value; BM SUV, mean: Bone marrow mean standardized uptake value; TNBC: triple-negative breast cancer; HER2: Human epidermal growth factor receptor-2.

To examine the risk effect of the Tumor-Infiltrating Lymphocyte index on mortality, a model was created using variables identified with p < 0.250 in univariate analyses and analyzed using the backward method. In this analysis, Stage 4 compared to Stage 2, HI3, HI2, BLR SUV mean increase was found to be risk factors, and SUVmin increase was found to be protective statistically significant factors (p < 0.01 p = 0.011 p = 0.043 p = 0.006).

**FIGURE 3:**
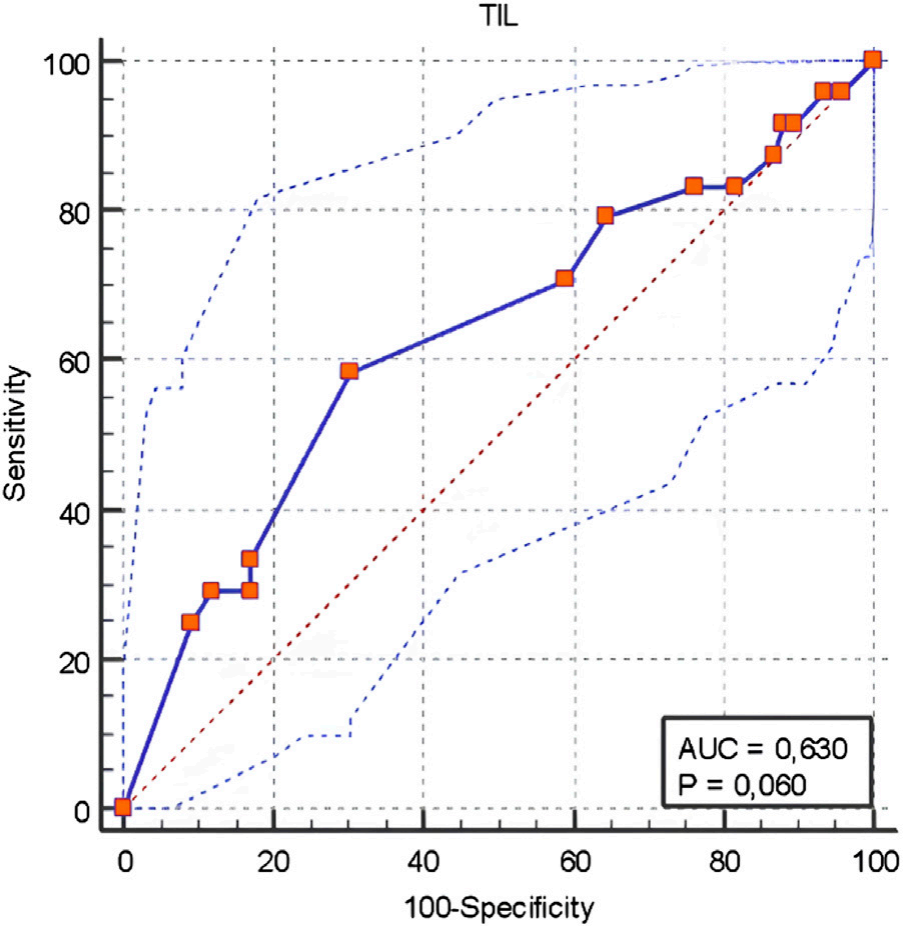
ROC Curve Analysis for the TIL cut-off value determining Stage 4.

The ROC curve analysis performed to determine the TIL cut-off value for Stage 4 determined as <5 with a sensitivity of 33.3%, specificity of 82.9%, positive predictive value of 38.1%, negative predictive value of 79.7%, and overall accuracy rate of 71% (Figure 3). The characteristics of patients with TIL levels of 0–10, 10–40, and >40 are shown in Table 7.

**TABLE 7:** Disease characteristics in TILs groups.

		TILs						p^a^
		0–10		10–40		>40		
		n	%	n	%	n	%	
TNBC/HER2	HER2	27	43.5	11	40.7	1	9.1	0.095
	TNBC	35	56.5	16	59.3	10	90.9	
Nuclear grade	2	3	4.8	2	7.4	0	0.0	0.804
	3	59	95.2	25	92.6	11	100.0	
T	1	1	1.6	1	3.7	0	0.0	0.713
	2	40	64.5	18	66.7	10	90.9	
	3	13	21.0	6	22.2	1	9.1	
	4	8	12.9	2	7.4	0	0.0	
N	0	17	27.4	7	25.9	2	18.2	0.166
	1	16	25.8	6	22.2	4	36.4	
	2	7	11.3	8	29.6	4	36.4	
	3	22	35.5	6	22.2	1	9.1	
M	0	45	72.6	23	85.2	9	81.8	0.397
	1	17	27.4	4	14.8	3	18.2	
Stage	2A	15	24.2	8	29.6	2	18.2	0.434
	2B	10	16.1	2	7.4	3	27.3	
	3A	5	8.1	7	25.9	3	27.3	
	3B	4	6.5	0	0.0	0	0.0	
	3C	11	17.7	5	18.5	1	9.1	
	4	17	27.4	5	18.5	2	18.2	
Multicentricity	negative	40	64.5	19	70.4	11	100	0.061
	Positive	22	35.5	8	29.6	0	0.0	
Axillary Lymph node	negative	17	33.9	7	33.3	2	18.2	0.581
	Positive	45	66.1	20	66.7	9	81.8	

^a^ Chi Square Test.

TILs, tumor-infiltrating lymphocytes; TNBC, triple-negative breast cancer; HER2, Human epidermal growth factor receptor-2; T, Tumor; N, lymph node; M, metastasis.

**FIGURE 4:**
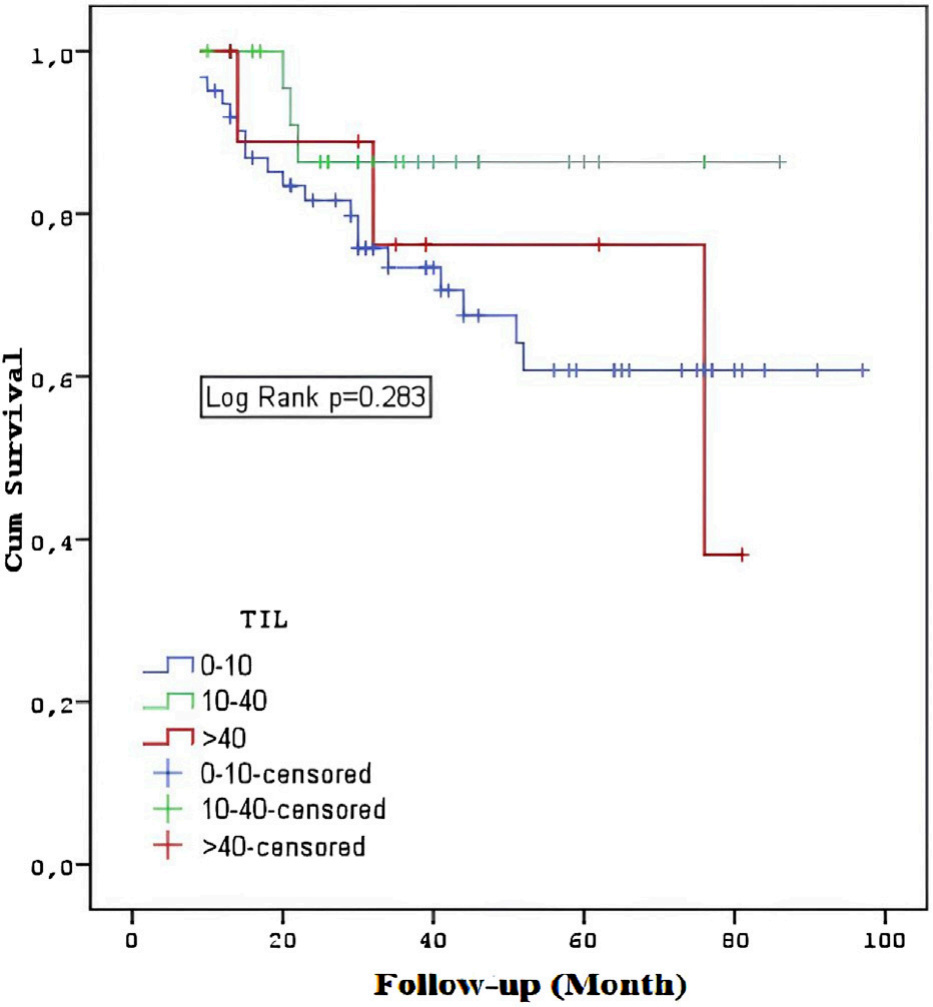
Overall survival among the TIL groups.

**FIGURE 5:**
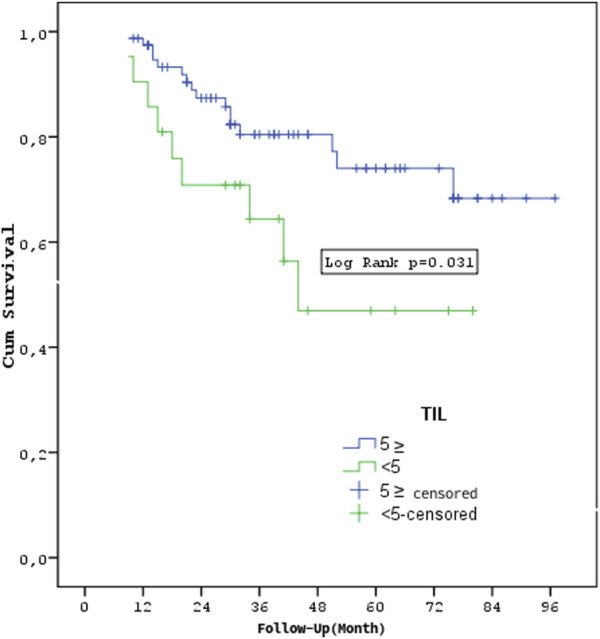
Survival according to TIL cut-off value.

Patients with stages T3-4 or M1 had a shorter overall survival rate (p = 0.033, p < 0.001, p < 0.001). The overall survival rates of the TIL groups did not differ substantially (p = 0.283) (Figure 4). In the TNBC and HER2 groups, there was no statistically significant difference in overall survival between the TIL groups (p = 0.322, p = 0.646). The survival rates of metastatic and non-metastatic patients in the TIL groups were not substantially different (p = 0.333, p = 0.786). Patients having a TIL cut-off value of <5 had significantly worse survival rates compared to those with a TIL value of ≥5 (p = 0.031) (Figure 5).

## Discussion

This study examined the relationship between tumor-infiltrating lymphocyte levels, FDG metabolic parameters, and tumor heterogeneity in non-luminal breast cancers and their relationship with overall survival. We found a significant relationship between TIL levels and FDG metabolic parameters in non-luminal breast cancers, especially in advanced-stage disease. Our data showed that TIL levels had moderate positive correlations with liver SUVmean, spleen SUVmax and SUVmean, and bone marrow SUVmax and SUVmean in Stage 4 patients. (p = 0.004, p = 0.007, p = 0.009). We also found that BLR SUV mean values were significantly higher in patients with short survival.

The role of TILs and ^18^F-FDG PET/CT metabolic characteristics in this advanced stage can shed light on the disease’s biology and potential therapeutic approaches. Higher TIL levels are linked to increased metabolic activity, implying that metabolic parameters could be useful prognostic markers [10, 11, 14, 22]. Further research is required on this topic.

Contrary to previous research by Murakami et al., which reported a positive correlation between ^18^F-FDG uptake and increased TIL levels indicated by higher SUVmax values, our study did not find a significant correlation between TIL levels and SUV values in non-luminal breast cancers [8]. Kitajima et al. also reported a positive correlation between TIL levels and SUVmax of FDG-PET in breast cancer patients, suggesting that SUVmax plays an important role in prognosis in less aggressive and immune-ineffective microenvironments such as SUVmax-low, TIL-low, or luminal subtype breast cancer [10]. Sasada et al. indicated that while both whole-body PET/CT and dedicated breast PET/CT could measure SUVmax to assess TIL levels, a high lymphocytic infiltrate was associated with higher SUVmax values only in dedicated breast PET, making it potentially more effective in evaluating the immune microenvironment, particularly in early-stage disease [23]. Similar findings were reported by Park et al., who noted a correlation between higher SUVmax values and higher TIL levels, and Hirakata et al., who also found a strong correlation between high TIL levels and high SUVmax [12, 24]. However, Kajary et al. reported no significant differences in kinetic parameters when comparing breast tumors with varying levels of stromal TIL, suggesting that stromal TIL does not significantly impact measured FDG kinetics [25].

Furthermore, new research has revealed spleen and bone marrow FDG uptake as critical biomarkers for determining the systemic immune response to malignancy. Increased FDG uptake in these organs may indicate an activated or stressed immune system, which is frequently associated with a poor prognosis due to systemic inflammation or immunological dysregulation, especially in advanced disease stages [11, 13–15]. Seban et al. have found that spleen glucose metabolism may be a stronger predictor of prognosis than bone marrow metabolism, with total metabolic tumor volume and spleen-to-liver glucose uptake ratio helping as independent prognostic indicators for predicting 5-year recurrence in breast cancer [11].

In our study, although no statistical correlation was observed between TIL levels and SUV values in non-luminal breast cancer, heterogeneity indices (HI2 and HI3) emerged as significant risk factors for mortality. This finding is consistent with previous studies highlighting the predictive value of FDG intratumoral heterogeneity in determining disease characteristics and survival outcomes across different cancer types [11, 16, 17, 26, 27]. Our results further revealed that TIL levels had a weak negative correlation with HI1 and HI3 in TNBC patients, while no correlation was observed in the HER2-positive group. Moreover, in stage 4 non-luminal breast cancer, TIL levels showed a moderately negative correlation with HI2 and HI3, indicating that higher TIL levels might correlate with less aggressive histological features even in advanced disease stages.

Interestingly, in our study, HI1, HI2, and HI3 values were found to be significantly higher in patients with short survival, while no statistically significant difference was found in survival rates with TIL groups (p = 0.283). In addition, no statistically significant difference was found in survival rates of TIL groups in TNBC and HER2 groups (p = 0.322 p = 0.646). This finding suggests a more complex relationship between TIL levels and patient outcomes. TIL may not be the sole determinant of survival, but it still plays a role in modulating tumor behavior and response to treatment [5, 10, 14, 15].

Previous research has shown that TIL, particularly when measured before chemotherapy, is related to a better prognosis and can predict the therapeutic impact of neoadjuvant chemotherapy in breast cancer [5, 8, 9]. However, in our investigation, patients with TIL levels less than 5 had significantly lower survival rates than those with TIL levels 5 or higher (p = 0.031), which is consistent with previous research suggesting that low TIL levels are associated with poorer outcomes across various breast cancer subtypes [6, 28].

This study’s limitations include its retrospective design, a small sample size from a single center, the use of biopsy samples for TIL assessment, classification by a single pathologist, and the lack of evaluation of treatments received or progression-free survival.

In conclusion, our study demonstrates a significant relationship between TIL, FDG metabolic parameters, and tumor heterogeneity in non-luminal breast cancers, especially in advanced stages. We found positive correlations between TIL levels and metabolic activity in critical organs like the liver, spleen, and bone marrow, which may serve as valuable prognostic indicators. Additionally, heterogeneity indices emerged as risk factors for mortality. These findings suggest that TIL levels and metabolic parameters offer deeper insights into tumor behavior and prognosis. Further research involving larger cohorts and diverse breast cancer subtypes is necessary to validate these findings and better understand the interplay between tumor metabolism and survival.

## Data Availability

The original contributions presented in the study are included in the article/supplementary material, further inquiries can be directed to the corresponding author.

## Abbreviations

NLBC, Non-luminal breast cancers; HER2, Human epidermal growth factor receptor-2; TNBC, Triple-negative breast cancer; TILs, Tumor-infiltrating lymphocytes; ^18^F-FDG, Fluorine-18 fluorodeoxyglucose; PET/CT, Positron emission tomography-computed tomography; T, Tumor; N, Lymph Node; M, Metastasis; SUVmax, Maximum standardized uptake value; SUVmin, Minimum standardized uptake value; SUVmean, Mean standardized uptake value; HI, Heterogeneity Index; MTV, Metabolic tumor volume; TLG, Total Lesion Glycolysis; SUVpeak, Peak standardized uptake value; SLR, Spleen-to-liver ratio; BLR, Bone marrow-to-liver ratio; IHC, Immunohistochemistry.
